# Assessing Somatic Symptoms With the Patient Health Questionnaire (PHQ-15) in Syrian Refugees

**DOI:** 10.1177/10731911221086986

**Published:** 2022-04-21

**Authors:** Pascal Schlechter, Jens H. Hellmann, Nexhmedin Morina

**Affiliations:** 1 University of Cambridge, UK; 2 University of Münster, Germany

**Keywords:** somatization, PHQ-15, measurement invariance, refugees, somatic symptoms

## Abstract

Somatic symptoms are common among Syrian refugees. To quantify somatic symptom load, sum score models derived from the Patient Health Questionnaire (PHQ-15) have been frequently applied without psychometric justification. Across two studies (total *N* = 776), we (a) tested different PHQ-15 factor solutions in Syrian refugees, (b) investigated measurement invariance (MI) of the factor solutions compared with German residents, and (c) scrutinized whether sum score models adequately represent the data and differ in associations with external validators compared with factor scores. One-factor, three-factor, four-factor, and a reduced one-factor solution all displayed acceptable to good model fit. The four-factor solution showed the best fit, enabling differential symptom analyses. Sum score models often had poor model fit, necessitating independent investigations before applying them. For all factor solutions, (partial) strict MI between residents and refugees could be established. All scoring methods displayed high and comparable associations with functional impairment, depressive, and anxiety symptoms.

More than 13 million Syrians have been forcibly displaced as a result of the Syrian civil war (UNHCR, 2020). Although most Syrian refugees fled to immediate neighboring countries, more than one million Syrian refugees have arrived in Germany since 2015 (UNHCR, 2020). Most of these individuals had been exposed to potentially traumatic events before and during their flight and remain vulnerable to psychological distress (Echterhoff et al., 2020). Meta-analytical findings suggest high levels of anxiety, depression, and post-traumatic stress disorder in refugees resettling in high-income countries (Henkelmann et al., 2020). However, clinical presentations and epidemiological research also highlight the role of somatic symptoms that remain medically unexplained among refugees (Hassan et al., 2015; Nesterko et al., 2020). In fact, somatic symptoms are common among Syrian refugees (Borho et al., 2021) and play a central role in their mental health symptom constellations (Schlechter, Wilkinson, et al., 2021).

Clinical studies with refugee populations often use sum score models derived from the Patient Health Questionnaire-15 (PHQ-15; Kroenke et al., 1998) to estimate somatic symptom distress (Nesterko et al., 2020). Although a limited range of psychometric aspects of the Arabic PHQ-15 have been explored with samples from university students (AlHadi et al., 2017) and primary care patients in Saudi-Arabia (Becker et al., 2002), the psychometric functioning of this version in Syrian refugee populations currently residing in Western receiving countries such as Germany remains largely unknown. Specifically, there is lack of knowledge regarding (a) the underlying factor structure in refugee populations, (b) measurement invariance (MI) compared with other populations, and (c) the validity of sum score models with external associations. The present investigation addressed these gaps to provide a sound psychometric basis for this important and practically relevant application of the PHQ-15.

## Psychometric Properties of the PHQ-15

Several research findings support the reliability, validity, and efficiency of this screening tool for somatization in populations in Western countries (Kroenke et al., 1998; van Ravesteijn et al., 2009; Zijlema et al., 2013). Furthermore, taxometric analyses support the dimensional structure of somatic symptoms assessed with the PHQ-15 (Jasper et al., 2012). Because of these desirable psychometric properties, the Diagnostic and Statistical Manual of Mental Disorders *5th edition (DSM-5)* workgroup suggested the PHQ-15 as a measurement tool of somatic symptom severity in somatic symptom disorders (*DSM-5*; American Psychiatric Association, 2013). Accordingly, researchers investigating refugee mental health in receiving countries commonly use sum score models to estimate somatic distress, implicitly assuming an underlying one-factor solution (Nesterko et al., 2020). Meanwhile, different factor solutions have been proposed for the PHQ-15 but not been systematically tested in refugee populations. A one-factor solution is the most pertinent solution to test because simple scale composite scores are convenient and often used (Nesterko et al., 2020). Such a factor solution was also suggested in a study that demonstrated good reliability and validity of the Arabic PHQ-15 in university students from Saudi-Arabia (AlHadi et al., 2017). Using the Chinese version of the PHQ-15 in other non-refugee populations, a three-factor solution was reported with the three correlated latent factors cardiopulmonary, gastrointestinal, and pain-fatigue symptoms (Liao et al., 2016; Zhang et al., 2016). A further differentiation was made with a four-factor solution by additionally separating the pain and fatigue factor in the general population and a sample of primary care patients in Germany (Witthöft et al., 2013; Zijlema et al., 2013). Recent advances reported an age and gender invariant bi-factor model that led to an increment in model fit compared with the other factor solutions in primary care patients in Spain (Cano-García et al., 2020). This model specifies one general somatization factor and four orthogonal specific factors from the four-factor solution mentioned above (Cano-García et al., 2020; Witthöft et al., 2013). Furthermore, in a representative sample of the general population in Germany, Gierk et al. (2014) applied a shortened Version of PHQ-15, the Somatic Symptom Scale-8 (SSS-8), consisting of eight items only. Although the proposed bi-factor structure reflects gastrointestinal, pain, fatigue, and cardiopulmonary factors alongside an orthogonal general somatic symptom factor, model fit for a one-factor solution was also acceptable (Gierk et al., 2014). Moreover, most studies that compared the SSS-8 against the PHQ-15 tested similarities in internal consistencies and construct validity of total scores of entire scales (Toussaint et al., 2017). In psychosomatic outpatients in Germany, internal consistencies, item total correlations, and associations with other constructs of the SSS-8 were comparable to those of the PHQ-15 (Gierk et al., 2015). As the SSS-8 is meant to be an abbreviated version of the PHQ-15 providing a parsimonious single score, a one-factor solution is the most pertinent to test in Syrian refugees.

## Measurement Invariance

To allow for unbiased mean level comparisons with other populations, multi-group invariance of the established factor solutions needs to be tested (Milfont & Fischer, 2010). Importantly, studies comparing the measurement properties of the PHQ-15 between certain populations revealed that the bi-factor model with a general somatization factor and four symptom-specific factors was comparable among German and Dutch samples, but not a Chinese sample (Leonhart et al., 2018). This demonstrates the relevance of independent testing because measurement models may be biased. Accordingly, MI of the PHQ-15 may be violated when the overall construct of somatization is differently represented in refugee populations, indicating a lack of conceptual equivalence between populations. Different factor solutions may represent a more appropriate reflection of the data in refugee samples compared with other populations. In fact, Syrian refugees have specific idioms to express distress, and these may be related differently to the construct of somatization (Hassan et al., 2015). Symptoms may thus be non-invariant if the used language fails to express them in a culturally appropriate way. Certain items may display floor effects when refugees hesitate to agree with statements that do not resonate with their cultural expression of somatic symptoms. This would decrease the ability of these items to differentiate between individuals on the latent factor and could be reflected in higher error variances of the items. There is a large variation in the way in which individuals interpret the meaning of language that can be influenced by slight nuances (Sulaiman et al., 2001). Accordingly, response categories may have different meanings for refugees than for members of the receiving countries leading to different item thresholds. For instance, cultural norms may impose more denial upon reporting problems among refugees because they fear being perceived as weak (Hassan et al., 2015). Refugees might thus be hesitant to admit that they are *bothered a lot* by certain symptoms and thresholds for this response option may differ compared with residential populations.

## Use of Sum Scores and External Validators

Congeneric factor models examining MI do not simply translate into sum score models in which symptoms equally contribute to the underlying latent construct (McNeish & Wolf, 2020). Items contain different information regarding the underlying construct by having different factor loadings. Equal treatment of items in composite scores is oversimplified and may lead to a loss of information. A simulation study suggests that 3% unexplained variability between factor scores and sum scores can result in different conclusions based on these scores (McNeish & Wolf, 2020). As counting somatization symptoms with the PHQ-15 is common practice, it is important to discern whether the factor models fit the data when all factor loadings of a given latent factor are constrained to be equal, which is the underlying assumption of sum scores. Furthermore, from a practical research perspective, it is relevant to test whether associations with external validators differ when factor scores are used compared with sum score models. This way, it is possible to examine the extent of potential bias in external associations when convenient sum score models are applied among refugee populations.

## The Present Research

We aimed to systematically examine the psychometric properties of the PHQ-15 in Syrian refugees residing in Germany (Witthöft et al., 2013; Zijlema et al., 2013). Our research goals were threefold. First, we examined model fit of different factor solutions in refugees: A one-factor solution with one latent factor, a three-factor solution with the correlated latent factors *pain-fatigue, gastrointestinal* and *cardiopulmonary* symptoms, a four-factor solution with the correlated latent factors *pain, gastrointestinal, cardiopulmonary*, and *fatigue*, a bi-factor model including the four orthogonal specific factors plus a general somatization factor, and the SSS-8 factor structure. Second, we investigated MI of these factor solutions in refugees compared with German residents to ensure that the same underlying construct is measured across groups. Third, we tested whether sum score models adequately represent the data and whether associations with external validators differ between factor scores and sum scores. External validators were based on commonly reported associations with somatic symptoms: Depressive symptoms, anxiety symptoms, and functional impairment (Jongedijk et al., 2020; Kohlmann et al., 2016; Nesterko et al., 2020).

## Method

### Participants and Design

Data were drawn from two studies investigating mental health in refugees in Germany compared with German residents (Schlechter, Kamp, et al., 2021). Respondents were at least 18 years old and provided informed consent. Participants were recruited via social media platforms and email lists, and took part online (monetary compensation: € 5). Ethical approval for data usage in both studies was granted by the psychology department’s ethics committee of the University of Münster. Participants provided informed consent to anonymize data usage for research purposes, including the examination of the current psychometric analyses. Sample sizes were guided by practical considerations based on the feasibility to collect data from refugees in Germany with a target of 200 participants per group (refugees and residents, respectively) for both studies. In Study 1, *N* = 402 individuals participated, of which 202 were German residents and 200 were refugees residing in Germany. Initially, *N* = 410 individuals participated in Study 2, of which 205 were German residents and 205 were refugees. In Study 2, participants were instructed to read a short text about mental health services and were subsequently asked to answer three content-related multiple choice questions to ensure attentive reading. Data from participants who provided false responses were excluded. We used established versions of the German questionnaires, and all scales were then translated into Modern Standard Arabic by a professional translation office. The translated versions were carefully double-checked and compared with the German versions by a native Arabic speaker who was fluent in both Arabic and German.

### Materials

#### PHQ-15

This instrument assesses the following somatization symptoms: *stomach pain, back pain, pain in arms and legs, pain during intercourse, headaches, chest pain, dizziness, heart race, short breath, constipation, nausea, gas, trouble sleeping*, and *feeling tired* (for a review of established versions of this questionnaire including the German version, see Kroenke et al., 2010). The latter two symptoms are taken from the PHQ-9 module assessing depressive symptoms. Symptoms are assessed for the last four weeks on a three-point Likert-type scale ranging from 0 (*not bothered at all*), over 1 (*bothered a little*), to 2 (*bothered a lot*). The derived total scores thus range from 0 to 30. Higher scores indicate greater severity of somatic symptoms. Cut-offs for somatic distress are Minimal (≤4), mild (≥5), moderate (≥10), and severe (≥15). The PHQ-15 was applied in both studies.

#### PHQ-9

In Study 1, the PHQ-9 was used as an external validator to assess depressive symptoms during the last 2 weeks (Spitzer et al., 1999). Response options range from *not at all* (0), *several days* (1), *more than half the days* (2), and *nearly every day* (3). The PHQ-9 (α_residents_ = .70; α_refugees_ = .81) showed acceptable to good internal consistencies in this study. The psychometric functioning of the PHQ-9 has been demonstrated across many populations (Gräfe et al., 2004; Kroenke et al., 2010, for a review) and in Middle Eastern refugees in Germany alongside evidence for scalar MI between refugees and residents (Schlechter, Wilkinson, et al., 2021). Because two items are included in the PHQ-15, we used PHQ-9 scale composite scores without these two items.

#### Functional Impairment

As an additional external validator, we used a question tapping into functional impairment with one PHQ item (Kroenke et al., 2010; Spitzer et al., 1999) (*If you checked off any problems, how difficult have these problems made it for you to do your work, take care of things at home, or get along with other people*?). Participants responded to this item on a scale from (1) *not difficult at all* to (4) *extremely difficult.*

#### PHQ-4

In Study 2, we used the PHQ-4 as external validator (Kroenke et al., 2009). It comprises four items on a four-point scale from 0 (*not at all*) to 3 (*nearly every day*). Items pertain to endorsement in the last two weeks referring to two core symptoms of depression and anxiety, respectively (Gräfe et al., 2004). MI of the PHQ-4 was established between German residents, migrants, and refugees living in Germany (Tibubos & Kröger, 2020).

### Analytic Strategy

#### Factor Solutions

Analysis code and data can be found in the open science framework (https://osf.io/c3t6h/?view_only=66c59c75fdd745f180e6ff15464b5795). Analyses were conducted in *R* (R Core Team, 2019) and we used the *lavaan* package for our factor analytical models (Rosseel, 2012). Because of the three response options of the PHQ-15, we treated data as categorical and used the weighted least squares mean and variance adjusted (WLSMV) estimator for all analyses (Asparouhov & Muthén, 2010). In both studies, we tested four different factor solutions as proposed by the literature with confirmatory factor analyses (CFA): A one-factor solution with one latent factor (AlHadi et al., 2017), a three-factor solution with the correlated latent factors *pain-fatigue, gastrointestinal* and *cardiopulmonary* (Zhang et al., 2016), a four-factor solution with the correlated latent *factors pain, gastrointestinal, cardiopulmonary*, and *fatigue* (Witthöft et al., 2013), and a one-factor solution of the SSS-8 (Gierk et al., 2015).^1^Table 2 shows the items that belong to each factor. The bi-factor model that we initially aimed to test did not converge indicating that the model is too complex for the data. Likewise, a bi-factor model with three orthogonal factors and one general somatization factor did not converge.^2^ For each of the factor solutions, we tested whether sum score models adequately represent the data by constraining all items of a given latent factor to be equal. Following criteria were used to evaluate model fit: The comparative fit index (CFI) and the Tucker–Lewis Index (TLI) should both be larger than 0.95 or 0.90, for a good or acceptable model fit, respectively (Hu & Bentler, 1999). For the standardized root mean square residuals (SRMR), values lower than 0.08 indicate acceptable fit (Hu & Bentler, 1999). For the root mean square error of approximation (RMSEA), values below 0.05 indicate good fit and values of 0.08 acceptable fit, and they should not exceed 0.10 (Browne & Cudeck, 1992). To have an estimation of reliability for both factor and sum score models, we estimated omega total ( $\omega_{total}$ ) (McDonald, 1999) and Cronbach’s α (Cronbach, 1951). Omega total considers the different factor loadings while Cronbach’s α assumes equivalent factor loadings; values > .80 indicate good internal consistency; values > .70 acceptable internal consistency (McNeish, 2018).

#### Measurement Invariance

To evaluate whether somatization can be compared between refugees and residents, we systematically tested MI between residents and refugees in a multigroup CFA framework (Meredith, 1993; Milfont & Fischer, 2010). To establish MI, increasingly constrained and nested models were sequentially tested against each other. In these hierarchically nested models, constraints are added at each step in addition to the former constraints (Meredith, 1993; see also Millsap, 2012). First, the factor structure was constrained to be equivalent across groups (configural invariance). This allows researchers to establish whether the construct is conceptually represented with the same factor structure across groups. Next, the factor loadings were constrained to be equal across groups to gauge whether the items relate to the somatization factor in the same way across groups in addition to having the same factor structure (weak/metric invariance). This level of MI allows to compare variances and covariances between the tested groups (Millsap, 2012). Then, item thresholds were additionally constrained to be equivalent to discern whether the observed thresholds conditional on the latent factor do not differ across groups in addition to having the same factor structure and equal factor loadings (strong/scalar invariance). In weighted least squares approaches (Muthén, 1984), item thresholds are introduced to account for the ordered nature of the observed data, assuming that participants’ responses reflect a discrete categorization of the underlying latent variable and that both are related by a threshold relationship. When an observed variable has *r* response categories, the variable has *r*−1 thresholds τ_j_ resulting in two thresholds for the three response options of the PHQ-15. Establishing this level of MI allows to compare the means, variances, and covariances of the latent factors between the groups (Millsap, 2012). Last, the residual variances of the items were also constrained to be equal to examine whether the amount of variance in the items not explained by the latent factor does not differ across groups in addition to having the same factor structure, equal factor loadings and thresholds (strict/residual invariance; Meredith, 1993). This level of MI indicates that group differences are truly attributable to differences in the underlying construct (Millsap, 2012). To test strict MI, theta instead of delta parameterization was used. To detect violations of MI, we evaluated changes (Δ) in the CFI and RMSEA. That is, we calculated the differences between the fit indices of two nested models and evaluated the level of their discrepancies with ΔCFI ≥ .010 and ΔRMSEA ≥ .007, indicating substantial deterioration in model fit (Chen, 2007; Meredith, 1993; Milfont & Fischer, 2010).^3^ We also report the $\chi^{2}$ —test difference test despite potentially leading to inflated Type 1 error rates, thus falsely indicating poor model fit (Sass et al., 2014). Non-invariance of certain items may not preclude the possibility of unbiased group comparisons (for a simulation study see Guenole & Brown, 2014). Few parameters (e.g., several factor loadings) can be relaxed in relation to the number of invariant parameters leading to relatively unbiased estimates (Byrne et al., 1989). Partial MI models were tested when full MI was not supported by iteratively freeing parameters according to their unconstraint between-group discrepancies (Byrne et al., 1989; Guenole & Brown, 2014). These models were then tested against the model established in the step before.

#### External Validation

We aimed to investigate the extent to which different derived PHQ-15 scores vary in their associations with external variables in the refugee sample. Given that MI between German residents and refugees from Syria was already established for the PHQ-9 (Schlechter, Wilkinson, et al., 2021) and the PHQ-4 (Tibubos & Kröger, 2020), we used sum scores of our external validators. For the PHQ-15, we extracted factor scores for each model with the empirical Bayes method (Muthén, 1998-2004). We compared the external associations of these factor scores with the sum scores of the respective models by using Pearson’s correlations. In Study 1, we compared the association of the different scoring methods with depressive symptoms (measured by the PHQ-9 excluding the two overlapping items) and functional impairment. In Study 2, we compared them with depressive and anxiety symptoms (PHQ-4). To have a comparison of maximally unconstrained associations, we used factor scores of the configural models and contrasted them to maximally constraint sum scores.

## Results

Demographic characteristics are depicted in Table 1. Of the 200 refugees in Study 1, 182 (91%) were Syrians. Because the present study focuses on Syrian refugees, we excluded data from 18 non-Syrian refugees from countries such as Afghanistan or Iraq, resulting in a final sample of *N* = 384. In Study 2, *n* = 11 refugees were not from Syria (e.g., again from Afghanistan or Iraq), and their data were excluded from further analyses. Data from seven further refugees were excluded because they failed the attention check. Hence, the final sample of Study 2 was composed of *n* = 187 refugees and *n* = 205 residents (total *N* for both studies = 776). More refugees were male; more residents were female in both studies, Study 1, χ^2^(1, *N* = 384) = 119.83, *p* < .001, and Study 2, χ²(1, *N* = 392) = 116.27, *p* ˂ .001. Also, the first refugee sample was younger than the second, *t* = -3.35, *p* ˂ .001.

**Table 1.** Demographic Characteristics of the Subsamples.

	Study 1		Study 2	
Demographic v﻿ariable	Syrian refugees (*n* = 182)	German residents (*n* = 202)	Syrian refugees (*n* = 187)	German residents (*n* = 205)
Age in years	*M* = 25.56 (*SD* = 9.19)	*M* = 28.13 (*SD* = 7.35)	*M* = 30.08 (*SD* = 9.19)	*M* = 26.32 (*SD* = 9.67)
Gender				
Male	146 (80%)	48	161 (86%)	65 (32%)
Female	36 (20%)	154	26 (14%)	140 (68%)
Reasons for leaving home country*				
War	157 (86%)	—	162 (87%)	—
Political persecution	94 (52%)	—	113 (60%)	—
Religious persecution	22 (12%)	—	31 (17%)	—
Violence	65 (36%)	—	72 (39%)	—
Economic reasons	25 (14%)	—	24 (13%)	—

*Note.* Overall *N* = 776. *SD* = standard deviation.

*Multiple reasons could be named.

### Descriptive Statistics

Descriptive statistics including the median and interquartile range of all PHQ-15 items from both studies can be found in Table 2. In the refugee samples, the item with the lowest endorsement was *fainting*, followed by *pain during intercourse* and *menstruation problems.* Although most items showed acceptable skewness and kurtosis, the symptom *fainting* was extremely non-normally distributed. This is in line with former studies, in which this item was excluded due to such distributions (Cano-García et al., 2020). Given that the PHQ-15 was never tested in Syrian refugees and the WLSMV estimator can handle non-normally distributed items (Asparouhov & Muthén, 2010), we tested the factor solutions in the refugee samples with and without this item. Also, in line with previous research (Cano-García et al., 2020), we excluded the symptom *menstruation problems* from our analyses because it is gender specific and both refugee samples were predominately male.

**Table 2:** Descriptive Statistics of PHQ-15 Items and Scale Composite Scores.

	Study 1						Study 2					
	Syrian refugees (*n* = 182)			German residents (*n* = 202)			Syrian refugees (*n* = 187)			German residents (*n* = 205)		
Symptom	*M/Med*	*SD/IQR*	*SK/Kurt*	*M/Med*	*SD/IQR*	*SK/Kurt*	*M/Med*	*SD/IQR*	*SK/Kurt*	*M/Med*	*SD/IQR*	*SK/Kurt*
1. Stomach pain (G)	0.53/0	0.68/1	0.88/−0.43	0.58/1	0.64/1	0.61/−0.61	0.66/1	0.70/1	0.57/−0.85	0.40/0	0.58/1	1.13/0.26
2. Back Pain (P)	0.69/1	0.73/1	0.56/−0.98	0.72/1	0.68/1	0.41/−0.85	0.74/1	0.75/1	0.46/−1.13	0.64/1	0.69/1	0.59/−0.78
3. Pain arm, legs (P)	0.74/1	0.81/1	0.51/−1.31	0.50/0	0.64/1	0.89/−0.31	0.73/1	0.70/1	1.41/−0.92	0.43/0	0.64/1	1.16/0.19
4. Menstruation	0.27/0	0.54/0	1.81/2.34	0.61/0	0.72/1	0.73/−0.76	0.37/0	0.67/1	1.55/0.94	0.35/0	0.61/1	1.53/1.16
5. Pain intercourse (G)	0.23/0	0.52/0	2.26/4.12	0.23/0	0.53/0	2.27/4.10	0.21/0	0.53/0	2.48/4.96	0.16/0	0.46/0	2.91/7.64
6. Headaches (P)	0.67/1	0.74/1	0.61/−0.98	0.88/1	0.68/1	0.15/−0.87	0.87/1	0.75/1	0.22/−1.19	0.67/1	0.64/1	0.41/−0.72
7. Chest pain (C)	0.49/0	0.68/1	1.02/−0.22	0.21/0	0.43/0	1.79/2.17	0.56/0	0.71/1	0.86/−0.59	0.19/0	0.43/0	2.11/3.70
8. Dizziness (C)	0.36/0	0.59/1	1.40/0.88	0.42/0	0.61/1	1.17/0.28	0.48/0	0.63/1	0.95/−0.18	0.34/0	0.58/1	1.53/1.27
9. Fainting	0.09/0	0.36/0	4.05/16.36	0.04/0	0.23/0	5.55/33.42	0.18/0	0.45/0	2.53/5.84	0.03/0	0.21/0	6.63/48.01
10. Heart race (C)	0.32/0	0.57/1	1.54/1.35	0.26/0	0.51/0	1.85/2.55	0.34/0	0.61/1	1.61/1.39	0.26/0	0.49/0	1.69/1.99
11. Short breath (C)	0.58/0	0.65/1	1.13/0.08	0.23/0	0.50/0	2.10/3.60	0.53/0	0.71/1	0.93/−0.46	0.18/0	0.46/0	2.65/6.35
12. Constipation (G)	0.58/0	0.69/1	0.77/−0.61	0.59/0	0.70/1	0.76/−0.67	0.59/0	0.71/1	0.77/−0.68	0.48/0	0.65/1	0.98/−0.16
13. Nausea, gas (G)	0.53/0	0.72/1	0.95/−0.47	0.67/1	0.69/1	0.52/−0.82	0.61/0	0.74/1	0.75/−0.80	0.52/0	0.66/1	0.90/−0.35
14. Trouble sleep (P/F)	1.49/1	1.03/1	0.18/−1.15	1.08/1	0.94/2	0.62/−0.47	1.33/1	0.99/1	0.50/−0.81	1.14/1	0.90/1	0.62/−0.26
15. Feeling tired (P/F)	1.54/1	0.99/1	0.19/−1.09	1.28/1	0.84/1	0.52/−0.22	1.53/1	1.01/1.5	0.25/−1.15	1.20/1	0.85/1	0.39/−0.40

*Note.* PHQ-15 = Patient Health Questionnaire-15; M = mean; Med = median; *SD* = standard deviation; IQR = interquartile range; SK = skewness; Kurt = kurtosis; C = cardiopulmonary, G = gastrointestinal, *p* = pain-fatigue symptoms (3 factor solution), *F* = fatigue symptoms (4 factor solution).

### Internal Consistencies

#### One-Factor Solution

Table 3 shows the internal consistencies of the factor solutions. For the one-factor solution, internal consistencies were good for refugees and residents in both studies (all values ≥ .82).

**Table 3:** Internal Consistencies for both Samples.

	Study 1				Study 2			
	*Syrian refugees* (*n* = 182)		*German residents* (*n* = 202)		*Syrian refugees* (*n* = 187)		*German residents* (*n* = 205)	
	*α*	$\omega_{total}$	*α*	$\omega_{total}$	*α*	$\omega_{total}$	*α*	$\omega_{total}$
1 *Factor*	.84	.85	.82	.83	.85	.85	.86	.86
3 *Factors*								
Cardiopulmonary	.69	.70	.65	.66	.72	.73	.66	.66
Gastrointestinal	.67	.70	.70	.72	.66	.70	.68	.71
Pain-fatigue	.74	.74	.63	.65	.73	.73	.64	.65
4 *Factors*								
Cardiopulmonary	.69	.70	.65	.66	.72	.73	.66	.66
Gastrointestinal	.67	.70	.70	.72	.66	.70	.68	.71
Pain	.71	.72	.65	.66	.60	.64	.60	.62
Fatigue	.68	.68	.70	.70	.77	.77	.70	.70
*SSS-8*	.79	.79	.71	.72	.79	.79	.75	.76

*Note.* α = Cronbach’s alpha; ω_total_ = omega total; SSS-8 = Somatic Symptom Scale-8.

#### Three-Factor Solution

In the three-factor solution, internal consistency was acceptable for all factors in the refugee sample according to $\omega_{total}$ in both studies. However, Cronbach’s α indicated non-acceptable fit for the gastrointestinal factor in both studies, and for the cardiopulmonary in Study 1 (all alphas ≥ .66). For residents, internal consistencies ranged from .63 (pain-fatigue, Study 1), and .73 (cardiopulmonary, Study 2).

#### Four-Factor Solution

In both studies, for the gastrointestinal and cardiopulmonary factor, we found the same pattern as for the three-factor solution. In Study 1, the latent pain factor was acceptable for refugees (≥ .71), but showed below acceptable internal consistency according to both indices for refugees in Study 2, and for residents in both studies (range .60 - .66). The fatigue factor had acceptable internal consistencies in Study 2 for refugees (.77 according to both indices) and for residents in both studies (all values ≥ .70), but had below acceptable internal consistency according to both indices for refugees in Study 1 (.68).

#### SSS-8 Factor

In both studies, internal consistencies were acceptable for both refugees and residents (range .71-.79).

### Factor Solutions

All factor solutions have been tested with the fainting item and without the fainting item (see Table 4). In all factor models, we observed a decrement in model fit according to the $\chi^{2}$ test-statistic and fit indices when the fainting item was included. Although this item had acceptable factor loadings (all λ ≥ .40) and model fit was still acceptable, it had the lowest factor loading in all solutions, which was also apparent in worse model fit in the sum score models. We, therefore, focus on the models without this item in the following analysis.

**Table 4.** Factor Solutions for the Syrian Refugee Samples Separately for Both Studies.

	Study 1 (*N* = 182)						Study 2 (*N* = 187)					
Factor s﻿tructure	$\chi^{2}(df)$	*p*	CFI	RMSEA	SRMR	TLI	$\chi^{2}(df)$	*p*	CFI	RMSEA	SRMR	TLI
1 Factor	143 (65)	˂.001	.967	.082	.101	0.961	153 (65)	˂.001	.962	.085	.100	0.955
1 Factor (fainting)	162 (77)	˂.001	.966	.082	.110	0.959	191 (77)	˂.001	.954	.090	.107	0.946
Sum score	216 (77)	˂.001	.942	.100	.130	0.941	226 (77)	˂.001	.935	.102	.122	0.934
Sum score (fainting)	252 (90)	˂.001	.934	.100	.144	0.934	285 (90)	˂.001	.923	.108	.131	0.922
3 Factors	104 (62)	.001	.982	.062	.094	0.978	92 (62)	.008	.987	.051	.090	0.984
3 Factors (fainting)	122 (74)	˂.001	.981	.060	.104	0.976	122 (74)	˂.001	.981	.057	.097	0.976
3 Factors sum	175 (72)	˂.001	.957	.089	.126	0.953	162 (72)	˂.001	.961	.082	.115	0.957
3 Factors sum (fainting)	201 (85)	˂.001	.949	.090	.141	0.946	208 (85)	˂.001	.951	.088	.122	0.948
4 Factors	73 (59)	.104	.994	.036	.083	0.992	71 (59)	.134	.995	.033	.085	0.993
4 Factors (fainting)	88 (71)	.074	.993	.037	.094	0.991	101 (71)	.011	.988	.048	.048	0.985
4 Factors sum	86 (68)	˂.001	.971	.073	.105	0.964	92 (67)	˂.001	.970	.077	.105	0.963
4 Factors sum (fainting)	109 (55)	˂.001	.965	.074	.120	0.958	124 (55)	˂.001	.962	.082	.112	0.954
SSS-8	40 (20)	.004	.977	.076	.084	0.968	43 (20)	.002	.976	.080	.078	0.966
SSS-8 sum	72 (27)	˂.001	.950	.096	.113	0.948	96 (27)	˂.001	.929	.117	.113	0.927

*Note.* df = degrees of freedom; CFI = comparative fit index; RMSEA = root mean square error of approximation; SRMR = standardized root mean square residuals; TLI = Tucker–Lewis index; SSS-8 = Somatic Symptom scale-8; (fainting) shows the factor solution including the fainting item.

#### One-Factor Solution

In both samples, the one-factor solution had good model fit according to CFI and TLI, but only close to acceptable model fit or non-acceptable model fit according to RMSEA and SRMR. The respective sum score models showed acceptable model fit according to CFI and TLI but not acceptable fit according to the other indices.

#### Three-Factor Solution

The three-factor solution had good model fit according to CFI and TLI, but only acceptable model fit according to RMSEA and close to acceptable fit according to the SRMR. Model fit was descriptively better than for the one-factor solution. The respective sum score model showed good fit according to CFI, TLI, acceptable fit according to RMSEA, but non-acceptable fit according to SRMR.

#### Four-Factor Solution

The four-factor solution had good model fit according to CFI, TLI, and RMSEA in both studies and non-acceptable fit according to the SRMR in Study 1, but acceptable SRMR fit in Study 2. Descriptively, it had the best model fit of the tested solutions and was also the only model with a non-significant $\chi^{2}$ test-statistics. The sum score model showed good model fit according to the CFI and TLI, acceptable fit according to RMSEA, and non-acceptable fit according to the SRMR.

#### SSS-8 Factor

The SSS-8 model had good model fit according to CFI and TLI and acceptable fit according to RMSEA in both studies and acceptable fit according to the SRMR only in Study 2. In Study 1, CFI and TLI indicated good model fit for the SSS-8 sum score model, RMSEA suggested acceptable fit and SRMR non-acceptable fit. In Study 2, CFI and TLI suggested acceptable fit for this model, but RMSEA and SRMR exceeded cut-offs for acceptable model fit.

### Measurement Invariance

Table 5 shows freely estimated factor loadings for both groups and both studies for the different factor solutions. Factor loadings for refugees were all good and exceeded .40. In Study 1, *pain during intercourse* displayed the lowest factor loadings in the refugee group, in Study 2, *headaches.* Factor loadings tended to be somewhat higher for their respective factors in the three- and four-factor solutions compared with the one-factor solution and the SSS-8. Some factor loadings were, however, problematic in the residents group. In Study 1, *pain in the arms and legs* had factor loadings that were below .30 for the one-factor solution. The same applied to *back pain* in Study 2. The largest factor loadings discrepancies between groups were also found for these symptoms.

**Table 5.** Freely Estimated Standardized Factor Loadings in the CFA.

	Study 1 Syrian refugees (*N* = 182)				Study 1 German residents (*N* = 202)				Study 2 Syrian refugees (*N* = 187)				Study 2 German residents (*N* =205)			
Symptom	1 F	3F	4F	SSS-8	1F	3F	4F	SSS-8	1F	3F	4F	SSS-8	1F	3F	4F	SSS-8
1. Stomach pain (G)	.66	.75	.75	.60	.52	.62	.62	.38	.67	.77	.77	.57	.68	.75	.75	.58
2. Back pain (P)	.54	.58	.66	.64	.33	.41	.42	.46	.62	.69	.77	.66	.29	.34	.40	.36
3. Pain arm, legs (P)	.73	.78	.91	.83	.29	.34	.52	.35	.60	.65	.74	.66	.39	.46	.54	.42
5. Pain intercourse (G)	.48	.52	.53	—	.38	.43	.43	—	.49	.51	.51	—	.54	.60	.59	—
6. Headaches (P)	.57	.61	.70	.58	.43	.51	.52	.43	.42	.46	.50	.40	.43	.48	.57	.44
7. Chest pain (C)	.56	.58	.58	.50	.59	.71	.72	.59	.66	.70	.70	.59	.65	.75	.75	.68
8. Dizziness (C)	.69	.71	.71	.71	.51	.64	.64	.51	.65	.69	.69	.65	.63	.75	.73	.70
9. Heart race (C)	.80	.82	.82	—	.57	.71	.71	—	.72	.76	.76	—	.59	.73	.68	—
10. Short breath (C)	.73	.76	.76	—	.61	.77	.77	—	.72	.76	.77	—	.71	.81	.81	—
11. Constipation (G)	.73	.82	.83	—	.77	.84	.84	—	.67	.76	.76	—	.78	.84	.84	—
12. Nausea, gas (G)	.72	.80	.80	—	.80	.91	.91	—	.73	.81	.81	—	.78	.85	.85	—
13. Trouble sleep (P/F)	.65	.68	.71	.66	.58	.69	.69	.67	.65	.70	.74	.72	.54	.65	.73	.60
14. Feeling tired (P/F)	.77	.81	.86	.76	.60	.79	.79	.74	.77	.85	.92	.82	.55	.66	.76	.63

*Note*. CFA = confirmatory factor analyses; 1F = one factor; 3F = three factors; 4F = four factors; SSS-8 = Somatic Symptom Scale-8; G = gastrointestinal; *p* = pain-fatigue symptoms (3 factor solution); C = cardiopulmonary; *F* = fatigue symptoms (4 factor solution).

#### One-Factor Solution

Table 6 shows the MI tests of the different factor solutions among refugees and German residents. Configural model fit was good to acceptable for the one-factor solution in Studies 1 and 2 according to CFI, RMSEA, and TLI, and non-acceptable according to SRMR. Model fit deteriorated substantially for the metric invariance model in both studies according to ∆CFI and ∆RMSEA. We could establish partial metric MI when setting factor loadings of *pain in arms and legs* free in Study 1, and factor loadings of *back pain* in Study 2. In Study 1, we could only establish partial scalar and strict MI when we additionally set the item thresholds of *headaches* (τ1_refugees_ = *−*0.01, τ2 _refugees_ = 1.18; τ1_residents_ = *−*0.59, τ2 _residents_ = 1.24) and *chest pain* (τ1_refugees_ = 0.33, τ2 _refugees_ = 1.51; τ1_residents_ = 1.10, τ2 _residents_ = 2.89) free. In Study 2, partial strict MI was established.

**Table 6:** Test of Measurement Invariance Between Syrian Refugees and German Residents.

Factor s﻿tructure	Study 1 (*N* = 384)								Study 2 (*N* = 392)						
	$\chi^{2}(df)$	CFI	RMSEA	SRMR	TLI	∆CFI	∆RMSEA		$\chi^{2}(df)$	CFI	RMSEA	SRMR	TLI	∆CFI	∆RMSEA
*1 Factor*								*1 Factor*							
Configural	323 (130)	.946	.088	.112	.935			Configural	321 (130)	.949	.087	.111	.939		
Metric*	383 (142)	.933	.094	.122	.926	.013	.006	Metric**	382 (142)	.936	.093	.117	.930	.013	.006
Metricp^a ns^	345 (141)	.941	.089	.118	.934	.005	.001	Metricp^d ns^	359 (141)	.942	.089	.114	.936	.007	.002
Scalarp***	422 (155)	.926	.095	.113	.926	.015	.006	Scalarp***	389 (155)	.938	.088	.112	.937	.004	.001
Scalarp^b^***	396 (151)	.932	.092	.113	.930	.009	.003	Strictp*	422 (168)	.932	.088	.113	.937	.006	.000
Strictp ^ns^	423 (164)	.928	.091	.118	.931	.004	.001								
*3 Factors*								*3 Factors*							
Configural	161 (124)	.987	.045	.087	.983			Configural	181 (124)	.985	.049	.092	.981		
Metric*	211 (134)	.978	.055	.097	.975	.009	.010	Metric ^ns^	202 (134)	.982	.051	.096	.051	.003	.002
Metricp^a ns^	189 (133)	.984	.047	.092	.982	.003	.002	Scalar***	226 (146)	.979	.053	.094	.977	.003	.002
Scalarp***	230 (145)	.976	.056	.088	.974	.008	.009	Strict*	255 (159)	.974	.056	.096	.975	.005	.003
Scalarp^b ns^	192 (141)	.984	.046	.088	.982	.000	.001								
Strictp ^ns^	222 (154)	.981	.048	.092	.981	.003	.002								
*4 Factors*								*4 Factors*							
Configural	139 (118)	.994	.031	.082	.992			Configural	142 (118)	.993	.033				
Metric ^ns^	169 (127)	.988	.042	.091	.985	.006	.009	Metric ^ns^	164 (127)	.990	.039	.091	.988	.003	.006
Metricp^a ns^	153 (126)	.992	.034	.087	.991	.002	.003	Scalar***	185 (138)	.987	.042	.089	.986	.003	.003
Scalarp*	166 (137)	.992	.033	.083	.991	.000	.001	Strict**	215 (151)	.983	.048	.091	.982	.005	.005
Strcitp **	201 (150)	.986	.042	.087	.985	.006	.009								
Strcitp^c ns^	182 (149)	.991	.034	.085	.990	.001	.001								
*SSS-8*								*SSS-8*							
Configural	64 (40)	.980	.057	.078	.972			Configural	84 (40)	.968	.075	.082	.956		
Metric**	97 (47)	.959	.075	.096	.951	.021	.022	Metric*	108 (47)	.956	.082	.095	.947	.012	.007
Metricp^a ns^	78 (46)	.974	.061	.086	.968	.006	.004	Metricp^d ns^	100 (46)	.960	.070	.092	.952	.008	.003
Scalarp***	125 (55)	.944	.082	.082	.943	.030	.021	Scalarp ^ns^	109 (55)	.961	.071	.087	.960	.001	.001
Scalarp^e^*	87 (51)	.971	.061	.080	.968	.003	.000	Strictp***	143 (63)	.948	.077	.093	.953	.009	.006
Strcitp***	120 (59)	.951	.074	.087	.953	.020	.013	Strictp^c ns^	120 (62)	.958	.070	.091	.962	.003	.001
Strcitp^f^*	99 (57)	.965	.063	.081	.966	.006	.002								

*Note.* The stars behind the model refer to the $\chi^{2}$ difference test with the less constraint nested less constrained model. df = degrees of freedom; CFI = comparative fit index; RMSEA = root mean square error of approximation; SRMR = standardized root mean square residuals; TLI = Tucker–Lewis index; ns = non-significant; Metricp = partial metric invariance; Scalarp = partial scalar invariance; Strictp = partial strict invariance.

a Setting loadings of Item 3 free. ^b^Setting thresholds of Items 6 and 7 free. ^c^Setting residuals of Item 7 free. ^d^Setting loadings of Item 2 free. ^e^ Setting thresholds of Items 1 and 6 free. ^f^Setting residuals of Items 1 and 7 free.

* *p* ˂ .05. **p ˂ .01. ***p ˂ .001.

#### Three-Factor Solution

In Study 1, we found the same pattern for the three-factor model with acceptable to good model fit for the configural model according to all indices. Partial strict MI could be established when setting free the same parameter as for the one-factor solution: Factor loadings of *pain in arms and legs* and item thresholds of *headaches* (τ1_refugees_ = *−*0.02, τ2 _refugees_ = 1.23; τ1_residents_ = *−*0.62, τ2 _residents_ = 1.70) and *chest pain* (thresold1_refugees_ = 0.34, τ2 _refugees_ = 1.54; τ1_residents_ = 1.21, τ2 _residents_ = 3.32). In Study 2, strict invariance was established with overall good to acceptable model fit.

#### Four-Factor Solution

For the four-factor solution, we found good model fit for the configural invariance model in both studies according to the CFI, RMSEA, TLI, and acceptable for according to the SRMR. In Study 1, again factor loadings of *pain in arms and legs* were set free to establish partial metric MI. Partial scalar MI was given. To establish strict partial MI with overall good model fit, we set the residuals of the symptom *headaches* free. In Study 2, we found strict MI with acceptable to good model fit.

#### SSS-8 Factor

Configural model fit was acceptable to good in both studies. Metric MI assumptions were violated in both studies. We thus set factor loadings of *pain in arms and legs* free in Study 1, and factor loadings of *back pain* in Study 2. To establish partial scalar MI in Study 1, we freed the thresholds of *stomach pain* (τ1_refugees_ = 0.22, τ2 _refugees_ = 1.57; τ1_residents_ = *−*0.01, τ2 _residents_ = 1.47) and *headaches* (τ1_refugees_ = *−*0.02, τ2 _refugees_ = 1.19; τ1_residents_ = *−*0.62, τ2 _residents_ = 1.08). To establish partial strict MI, we relaxed constraints on the residuals of *stomach pain* and *chest pain* in Study 1 and *chest pain* in Study 2.

### Mean Differences

Establishing these levels of MI allows a relatively unbiased interpretation of the unadjusted manifest mean differences reported in Table 7 (Guenole & Brown, 2014). To ensure that this assumption holds, we also report the latent mean differences of the models with the highest constraints, that is partial strict MI or strict MI (see Table 7). In Study 1, refugees reported higher unadjusted manifest mean levels in the SSS-8 solution, higher levels of *pain-fatigue* symptoms on the three-factor solution, and higher values on the latent factors *cardiopulmonary* and *fatigue* symptoms in the four-factor solution. In Study 2, refugees reported higher values on all latent factors. The latent mean differences yielded the same interpretations with the exception that residents reported slightly higher means on the *pain* factor in the four-factor solution in Study 1.

**Table 7.** Descriptive Statistics of Scale Composite Scores and Internal Consistencies for Refugee Samples.

	Study 1							Study 2						
	*Syrian Refugees* (*n* = 182)		*German Residents* (*n* = 202)					*Syrian Refugees* (*n* = 187)		*German Residents* (*n* = 205)				
Factor s﻿tructure	*M*	*SD*	*M*	*SD*	*t*	*g*	*Latent* *Diff.*	*M*	*SD*	*M*	*SD*	*t*	*g*	*Latent* *Diff.*
*1 Factor*	9.00	5.92	8.30	4.61	−1.28	−0.13	0.11	9.73	6.18	6.99	4.38	−5.02***	−0.52	0.49***
*3 Factors*														
Cardiopulmonary	1.71	1.72	1.47	1.34	−1.56	−0.16	0.22	2.04	1.90	1.19	1.44	−5.00***	−0.51	0.73***
Gastrointestinal	1.87	1.89	2.07	1.88	1.07	0.11	−0.16	2.07	1.93	1.56	1.72	−2.74**	−0.28	0.38**
Pain-fatigue	5.13	3.02	4.46	2.39	−2.39*	−0.25	0.36**	5.20	2.94	4.08	2.26	−4.20***	−0.43	0.38***
*4 Factors*														
Cardiopulmonary	1.71	1.72	1.47	1.34	−1.56	−0.16	0.16	2.04	1.90	1.19	1.44	−5.00***	−0.51	0.73***
Gastrointestinal	1.87	1.89	2.07	1.88	1.07	0.11	−0.16	2.07	1.93	1.56	1.72	−2.74**	−0.28	0.38**
Pain	2.09	1.82	2.10	1.31	−0.31	0.01	−0.32*	2.34	1.64	1.75	1.34	−4.20***	−0.40	0.39**
Fatigue	3.04	1.76	2.36	1.54	−4.02***	−0.41	0.43***	2.86	1.81	2.33	1.50	−3.13**	−0.32	0.33**
*SSS-8*	6.52	4.02	5.67	3.03	−2.33*	−0.24	0.33**	6.91	4.00	5.01	2.98	−5.29***	−0.54	0.54***

*Note.* M = mean; *SD* = standard deviation; *t* = t-value; g = hedges’ g; Latent diff = latent difference for the factor model with the highest constrains (including partial measurement invariance); SSS-8 = Somatic Symptom Scale-8.

* *p* ˂ .001. **p ˂ .01. ***p ˂ .001.

### External Validation

#### Correlations Among Scoring Methods

In Study 1, the correlations between sum scores and their respective factor scores ranged from *r* = .86 (for the cardiopulmonary factor for both factor solutions) to *r* = .99 (for the one-factor solution and the SSS-8 solution). Between 2% and 26% of the variance between the scores remained thus unexplained. In Study 2, the correlation between sum scores and their respective factor scores ranged from *r* = .90 (for the pain factor for the four-factor solutions) to *r* = .99 (for the one-factor solution and the SSS-8 solution). Here, between 2% and 19% of the variance remained unexplained.

#### External Associations

All derived scores correlated highly with *functional impairment*, and *anxiety* and *depressive* symptoms (see Table 8 for correlations and 95% CIs). For the one-factor solution and the SSS-8 factor solution, sum scores and factor scores did not differ in their associations with external variables. In the three-factor solution, the *pain-fatigue* factor scores and sum scores showed the highest associations with outcome measures and the different scoring methods did not differ from each other. For the *gastrointestinal* and the *cardiopulmonary* symptoms, lower associations were found descriptively compared with the *pain-fatigue* factor. Moreover, sum scores and factor scores deviated from each other, although most confidence intervals still overlapped. Factor scores descriptively displayed higher correlation than sum scores. For the four-factor solution, the *fatigue* scores had the highest association with external associations. Sum scores and factor scores did not deviate from each other. The other three factors displayed lower associations and the sum scores deviated from the respective factor scores with deviations of up to .17. For the *cardiopulmonary* symptom factor and its association with the PHQ-9 in Study 1, sum score and factor score intervals did not overlap.

**Table 8.** Pearson’s Correlations of Factor Scores and Sum Scores of the Different Factor Solutions With the PHQ-9, Functional Impairment and PHQ-4 for the Syrian Refugee Sample.

	Study 1		Study 2	
Factor structure	PHQ-9	Functional impairment	PHQ-4 Depression	PHQ-4 Anxiety
*1 Factor solution*				
Sum scores	.72 [.65; .72]	.54 [.43; .64]	.55 [.44; .64]	.64 [.54; .72]
Factor scores	.72 [.64; .78]	.54 [.43; .64]	.54 [.43; .64]	.63 [.53; .71]
*3 Factor solution*				
Pain-fatigue sum scores	.72 [.65; .79]	.50 [.38; .60]	.55 [.44; .64]	.56 [.45; .65]
Pain-fatigue factor scores	.73 [.66; .80]	.54 [.42; .63]	.56 [.46; .65]	.62 [.53; .70]
Gastrointestinal sum scores	.51 [.39; .61]	.45 [.32; .56]	.36 [.23; .48]	.47 [.36; .57]
Gastrointestinal factor scores	.66 [.56; .73]	.53 [.41; .53]	.48 [.36; .58]	.57 [.47; .66]
Cardiopulmonary sum scores	.56 [.44; .65]	.42 [.29; .53]	.44 [.32; .55]	.57 [.47; .66]
Cardiopulmonary factor scores	.72 [.64; .78]	.54 [.43; .63]	.53 [.42; .63]	.63 [.53; .71]
*4 Factor solution*				
Pain sum scores	.46 [.34; .57]	.33 [.19; .45]	.35 [.22; .47]	.39 [.26; .50]
Pain factor scores	.63 [.54; .71]	.47 [.34; .57]	.50 [.38; .60]	.57 [.46; .66]
Gastrointestinal sum scores	.51 [.39; .61]	.45 [.32; .56]	.36 [.23; .48]	.47 [.36; .57]
Gastrointestinal factor scores	.66 [.57; .73]	.53 [.41; .62]	.48 [.36; .58]	.57 [.47; .66]
Cardiopulmonary sum scores	.56 [.45; .65]	.42 [.29; .53]	.44 [.32; .55]	.57 [.47; .66]
Cardiopulmonary factor scores	.73 [.66; .79]	.55 [.43; .64]	.53 [.42; .63]	.63 [.53; .71]
Fatigue sum scores	.77 [.70; .82]	.52 [.41; .62]	.57 [.46; .66]	.56 [.46; .66]
Fatigue factor scores	.76 [.69; .82]	.55 [.44; .65]	.59 [.49; .68]	.64 [.55; .72]
*SSS-8*				
Sum scores	.74 [.67; .80]	.55 [.44; .64]	.58 [.48; .67]	.63 [.54; .71]
Factor scores	.73 [.65; .79]	.54 [.42; .63]	.57 [.47; .66]	.63 [.54; .71]

*Note.* Numbers in brackets indicate 95% confidence intervals. All correlations were significant at *p* ˂ .001. PHQ = Patient Health Questionnaire; SSS-8 = SSS-8 = Somatic Symptom Scale-8.

## Discussion

Using data from two studies, we examined the psychometric properties of the PHQ-15 in Syrian refugees currently residing in a Western receiving country (i.e., Germany). The symptom *fainting* had the lowest endorsement and led to a decrement in model fit when it was included. Although including this item may not lead to drastically worse model fit, this item may lead to lack of accuracy especially in sum score models, given that it contributes to more heterogeneous factor loadings. In line with previous research, we thus excluded the item from further analyses (Cano-García et al., 2020). However, we need to acknowledge that this item might be theoretically relevant and future studies including this item are advised to conduct independent testing in their samples.

The one-factor solution had adequate model fit. Sum score models, too, had acceptable fit according to some fit indices, while the RMSEA and SRMR mainly indicated poor model fit for sum score models. Omega total and Cronbach’s α indicated good internal consistencies for the one-factor solutions. The three-factor and four-factor solutions fitted the data descriptively better with the latter solution displaying the best model fit, in line with psychometric studies in the general population as well as primary care patients in Germany and China (Liao et al., 2016; Witthöft et al., 2013; Zhang et al., 2016; Zijlema et al., 2013). The respective sum score model seemed acceptable according to some fit indices. The SSS-8 model showed also good to acceptable model fit. Consistent with findings in psychosomatic outpatients in Germany, the psychometric properties were comparable to the PHQ-15 (Gierk et al., 2015). In principle, all models seem applicable in line with the often-reported good psychometric properties of the PHQ-15 (Zijlema et al., 2013). The present study thus extends previous research on the psychometric functioning of the Arabic PHQ-15 version to Syrian refugees currently residing in Germany, while it was formerly tested with university students in Saudi-Arabia (AlHadi et al., 2017).

The different models have their own merits. The one-factor solution allows for convenient applications, for instance, in large epidemiological studies or in a multistep diagnostic process, where single total scores with cut-off values are practical. In case resources are limited further, the SSS-8 model can be used, in line with its initial purpose (Gierk et al., 2014). The three-factor and four-factor solutions may allow for fine-grained analyses such as elucidating distinct symptom profiles. Some evidence for this use is supported by our finding that the subscales showed differential associations with external validation measures. In the three-factor solution, the *pain* factor showed the strongest associations with external validators. However, this was likely attributable to the *fatigue* items because the *fatigue* factor showed the strongest associations with external validators when tested separately in the four-factor solution while associations of the *pain* factor became weaker. The *cardiopulmonary* and *gastrointestinal* symptom factors may display higher correlations with other constructs not assessed in the present studies (e.g., panic symptoms).

External sum score associations differed more strongly compared with factor scores for the three-factor and four-factor solutions. For instance, for the *cardiopulmonary* symptom factor the associations with depressive symptoms differed for the two scoring methods. Correlations between the factor scores and sum scores were also lower for the subfactor compared with the entire scale. This evidence aligns with simulation studies that suggest unexplained variability between sum and factor scores can lead to different conclusions (McNeish & Wolf, 2020). In the present studies, sum scores appeared to be slightly downwardly biased, which should be considered by researchers applying fine-grained symptom analyses with these scales. Although some information is lost according to worse model fit of this solution, we did not detect these practical problems concerning external associations for the one-factor solution.

Recent studies tested more granulated bi-factor models that did not converge in our own analyses (Cano-García et al., 2020; Leonhart et al., 2018). The models may have been too complex for our data given sample size restrictions. However, this may be a problem that researchers face in refugee studies because of typically small sample sizes due to practical constraints (Borho et al., 2021). It, thus, remains open whether a general somatization factor in a model that additionally accounts for the variance by specifying specific orthogonal factors reduces biases in total scores meaningfully. It should be established in further studies whether a potential reduction of bias through such models outweighs their computational complexity.

We established either partial or full strict MI between Syrian refugees in Germany and German residents for the tested models, which provides evidence that mean level comparisons between these two groups can be made (Milfont & Fischer, 2010). Previous work has demonstrated comparable scale properties in German and Dutch samples, but not in a Chinese sample (Leonhart et al., 2018). In addition, MI was demonstrated for gender and age in previous studies (Cano-García et al., 2020; Gierk et al., 2014). Demonstrating MI between refugees and members of the residential population is an important premise for understanding cross-cultural differences in somatic symptom prevalence. When problems with metric MI were encountered, this was mainly attributable to lower factor loadings in the German subsample. Therefore, these items seem not to be problematic in refugees. Concerning scalar MI, we found different thresholds between refugees and residents for a few items. Although the thresholds for *headaches* were lower in residents, the thresholds for *chest pain* were considerably lower in refugees. Also, the thresholds for *stomach pain* were higher for refugees in one model. It needs to be noted that there is no clear consensus on the number of parameters that can be freed to achieve partial MI (for a review see Putnick & Bornstein, 2016). Some studies suggest that at least half of the parameters should be invariant (Vandenberg & Lance, 2000) while simulation studies demonstrate increasing mean level bias with increasing non-invariant items (Guenole & Brown, 2014). We set a maximum of two thresholds per model free, and a maximum of four parameters in total, constituting a relatively small proportion of the estimated parameters. Only in the four-factor solution in Study 1, one tested mean had a different result when manifest unadjusted scores were compared with partially invariant latent scores.

### Limitations

It must be noted that the present findings reflect a limited perspective. This is because Syrian refugees have specific idioms to describe somatic symptoms and it remains unknown whether the PHQ-15 captures them adequately (Hassan et al., 2015). It may be that other symptoms or other linguistic subtleties that resonate better with these idioms may constitute a more accurate latent somatization factor. Future studies should include additional items that are specifically tailored to Syrian refugees’ socio-cultural background. Qualitative studies and item development involving people with lived experience are needed. Also, Modern Standard Arabic was used. Although the translations were double-checked by a native speaker, several parallel forward translations and back-translations by independent bilingual and bicultural translators would be the most accurate way of translating scales in cross-cultural research (Cha et al., 2007). In addition, testing other regional Arabic dialects may unravel nuanced language differences. Understanding such subtleties may prove relevant for practitioners’ readiness to work with refugees (Schlechter, Hellmann, et al., 2021). The present studies used convenience samples, hence cross-validation in different samples is warranted. Gender distribution was unequal between refugees and residents, and we lacked the power to test invariance of gender in addition to the cultural background. Future studies should empirically test whether gender contributes to measurement variations in somatization. For instance, *pain during intercourse* had relatively low factor loadings compared with other symptoms, which could be attributable to gender-specific factors. Although the PHQ-15 was invariant across gender in a large sample of outpatients in Spain (Cano-García et al., 2020), this needs to be independently tested with Syrian refugees. This is because Syrian refugees may have different gender norms than other populations, which may influence the articulation and expression of somatic symptoms (Hassan et al., 2015). Although the sample distribution of predominantly young men mirrors the sociodemographic composition of Syrian refugees in Germany (Juran & Broer, 2017), it needs to be established whether somatic symptoms can be unbiasedly assessed across gender.

Both samples could also have been combined to have more statistical power. However, there was a period of a few months between the two data collections and researchers have highlighted the importance of time-varying differences depending on the migration stage (Wu et al., 2021), based on which we decided to test the samples independently. Moreover, we needed to keep the samples separate for the external validation analysis and wanted to ensure that the tested models reflect the data adequately in these specific samples. Also, the first refugee subsample was somewhat younger than the second subsample, and testing the factor solutions with smaller samples mirrors sample sizes of common convenience samples in refugee research.

For our model evaluation, we used cut-off values that have been commonly applied to determine the fit of CFAs (e.g., Browne & Cudeck, 1992; Hu & Bentler, 1999). However, these cut-offs are based on maximum likelihood estimation and may not discover misfit of the model when using the WLSMV estimator for categorical data (e.g., Xia & Yang, 2019). Our model fit conclusions and derived recommendations need thus to be carefully interpreted when using the PHQ-15 in Syrian refugees by considering different aspects of applicability and model fit. Establishing MI with categorical data has limitations. Using $\chi^{2}$ —test statistics may lead to inflated Type 1 error rates, especially with large sample sizes, thus falsely indicating poor model fit (Sass et al., 2014). The validity of cut-off values for fit indices has however not been conclusively examined. This again points to the need of cross-validation.

Demonstrating (partial) strict invariance provides evidence for the internal validity of the PHQ-15. Yet, we did not have a broad range of external measures regarding mental health outcomes to prove external validity. Having a broader nomological network could unravel whether the PHQ-15 captures the entire breadth and complexity of the somatization construct. In this regard, it is also noteworthy that functional impairment was assessed with only one item. Area under the curve studies are warranted to demonstrate how well the PHQ-15 discriminates against clinical interviews to establish diagnoses. In addition, there was no attention check to ensure attentive participation in Study 1.

## Conclusion

The current research sheds light on the applicability of the PHQ-15 in Syrian refugee populations who fled to Germany. It adds evidence regarding the scale’s good psychometric functioning of different factor solutions and the potential use of sum scores. However, for the most accurate assessment of somatic symptoms in Syrian refugees, using factor scores is advisable because sum score models had the worst overall model fit. In sum, our data support the validity of the PHQ-15 in assessing somatic symptoms in Syria refugees with a history of potentially traumatic events and with different cultural backgrounds than most of the previously studied populations.

## Notes

1 The original SSS-8 differs from the PHQ-15 in its response format by using a five-point Likert-type scale (Gierk et al., 2014).

2 These models also encountered convergence problems when we combined the samples to have more power (some estimated variances were negative).

3 There is no clear consensus on these cut-offs for categorical data and we thus used these rather strict cut-offs. The X²-test statistic was not deemed suitable because it likely inflates minor differences when a sample size is sufficiently large.
